# Maternal immune activation primes deficiencies in adult hippocampal neurogenesis

**DOI:** 10.1016/j.bbi.2021.07.021

**Published:** 2021-10

**Authors:** Amalie C.M. Couch, Thomas Berger, Bjørn Hanger, Rugile Matuleviciute, Deepak P. Srivastava, Sandrine Thuret, Anthony C. Vernon

**Affiliations:** a Department of Basic and Clinical Neuroscience, Institute of Psychiatry, Psychology and Neuroscience, King’s College London, London, UK; b MRC Centre for Neurodevelopmental Disorders, King’s College London, London, UK

**Keywords:** Maternal immune activation, Prenatal insult, Hippocampus, Neurogenesis, Stress, Psychiatric illness, Neurodevelopmental disorders, Human induced pluripotent stem cells

## Abstract

Neurogenesis, the process in which new neurons are generated, occurs throughout life in the mammalian hippocampus. Decreased adult hippocampal neurogenesis (AHN) is a common feature across psychiatric disorders, including schizophrenia, depression- and anxiety-related behaviours, and is highly regulated by environmental influences. Epidemiological studies have consistently implicated maternal immune activation (MIA) during neurodevelopment as a risk factor for psychiatric disorders in adulthood. The extent to which the reduction of hippocampal neurogenesis in adulthood may be driven by early life exposures, such as MIA, is however unclear. We therefore reviewed the literature for evidence of the involvement of MIA in disrupting AHN. Consistent with our hypothesis, data from both *in vivo* murine and *in vitro* human models of AHN provide evidence for key roles of specific cytokines induced by MIA in the foetal brain in disrupting hippocampal neural progenitor cell proliferation and differentiation early in development. The precise molecular mechanisms however remain unclear. Nonetheless, these data suggest a potential latent vulnerability mechanism, whereby MIA primes dysfunction in the unique hippocampal pool of neural stem/progenitor cells. This renders offspring potentially more susceptible to additional environmental exposures later in life, such as chronic stress, resulting in the unmasking of psychopathology. We highlight the need for studies to test this hypothesis using validated animal models of MIA, but also to test the relevance of such data for human pathology at a molecular basis through the use of patient-derived induced pluripotent stem cells (hiPSC) differentiated into hippocampal progenitor cells.

## Highlights

• Reduced AHN is linked closely with risk and resilience for psychiatric illness.
• Prenatal MIA exposure reduces AHN through direct and indirect mechanisms.
• We hypothesise that AHN may mediate susceptibility or resilience to MIA.
• Studies in animal and human cellular models are required to test this hypothesis.

## 1 Introduction

Historically adult hippocampal neurogenesis (AHN) is a strongly contested topic. The concept of human AHN was first mooted in the mid-1960′s, with subsequent publications providing evidence for the generation of new neurons in the mammalian hippocampus that continues throughout adult life, but declines with age (Kuhn et al., 2018, Altman and Das, 1965). The first evidence for human AHN was provided by Eriksson et al. in 1998. Human brain tissue from patients which were administered the thymidine analog, bromodeoxyuridine (BrdU) was analysed. BrdU is incorporated into DNA during the S-phase. The authors identified neuronal cells that were positive for BrdU indicating that these must have originated from progenitors after the administration of BrdU, indicating the generation of new neurons in the adult hippocampus (Eriksson et al., 1998). Later, Spalding and colleagues used a carbon dating method to estimate the number of newly generated granule neurons at roughly 700 cells per day (Spalding et al., 2013). Recently, the existence of AHN in humans has been under debate after the publication of a study investigating *post-mortem* hippocampal tissue across a large age range. The authors reported that while they were able to detect newborn neurons in the tissue of young brains, their numbers drop to undetectable levels in adulthood, putting a large questionmark behind the role of AHN in humans (Sorrells et al., 2018). Shortly after this publication, several groups published results contrasting those of Sorrells and colleagues providing evidence for immature neurons and therefore AHN in post-mortem tissue of patients even in their 70 s and 80 s (Tobin et al., 2019, Moreno-Jiménez et al., 2019, Boldrini et al., 2018). These differences in observation of neurogenic markers may arise from several factors, including differences in tissue preparation, or medical backgrounds of patients as summarised elsewhere (Kempermann et al., 2018). Most recently, another publication by Sorrells et al. systematically brought together data from existing studies and added experiments to show that common markers for immature neurons, such as doublecortin (DCX), may not necessarily only label immature neurons, concluding that AHN, if present at all, may be extremely rare in adults. In response María Llorens-Martín, one of the authors identifying AHN in tissue from older people, pointed out weaknesses in this study, questioning Sorrells claim of neglectable levels of AHN (Sorrells et al., 2021). Taken together, human adult AHN, is still under heavy debate. More studies and refined techniques may be needed to settle this debate once and for all (Lee and Thuret, 2018). Nevertheless, current evidence indicating the existence of adult human AHN outweighs evidence for a negligible role of newborn neurons in the hippocampus.

**Fig. 1:**
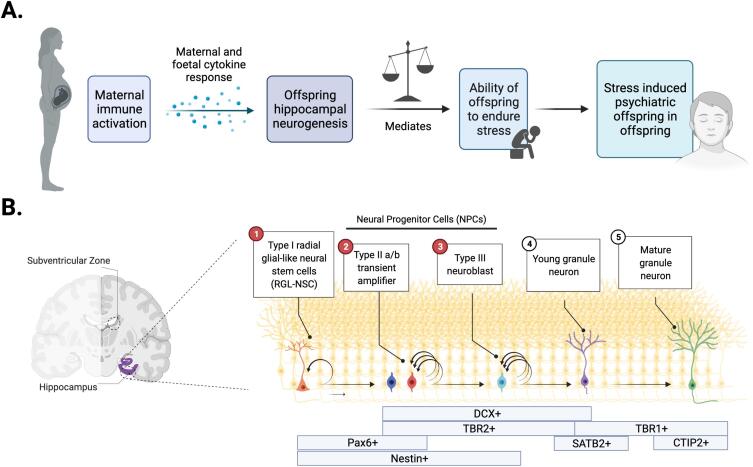
Theoretical framework for understanding the interplay between AHN and MIA. A) The potential for MIA to increase susceptibility to psychiatric illness in offspring, as mediated by hippocampal neurogenesis. Each factor (maternal immune activation, hippocampal neurogenesis and stress) confers the offspring’s resilience or susceptibility to the next B) Background and cell timeline adapted from (Berger et al., 2020) the molecular and cellular understanding of adult hippocampal neurogenesis, with the relevant cell marker expressions in boxes below. Stages 2 and 3, depicting type II a/b transient amplifier and type III neuroblasts are considered neural progenitor cells. Together with the first stage, type I radial glial-like neural stem cells, and denoted by the stages coloured red, these three neural stem / progenitor cell stages are particularly vulnerable to the effects of MIA-associated stimuli. Besides the subgranular zone, the subventricular zone is highlighted as well, representing the other well studied region of adult neurogenesis. Graphics created with BioRender.com. (For interpretation of the references to colour in this figure legend, the reader is referred to the web version of this article.)

Accepting that debate still continues regarding adult human AHN, the mammalian hippocampus and AHN are critically involved in regulating several domains of human and animal behaviour (Abrous et al., 2021). The on-going shaping of hippocampal circuitry throughout life by the process of AHN has been attributed to different forms of learning and memory (Dupret et al., 2008, Sasaki et al., 2018), cognitive abilities such as pattern separation and cognitive flexibility, emotional regulation and response to stress (Toda et al., 2019, Bartsch and Wulff, 2015). Within the context of this review, stress is defined as the psychological and biochemical demands induced by environmental factors that evoke an individual’s adaptive behaviour (Syed and Nemeroff, 2017). It is therefore not surprising to find evidence for the role that AHN plays in conferring risk or resilience to serious human mental illnesses (Fig. 1a), as previously reviewed elsewhere (Kempermann et al., 2008, Kang et al., 2016). Evidence from animal and human *post-mortem* brain tissue studies provide evidence for reductions in AHN and an immature dentate gyrus as one potentially causal basis for the increased risk of psychiatric disorders associated with exposure to stress, including depression, anxiety and schizophrenia (SZ) (Kang et al., 2016, Anacker, 2014, Yamasaki et al., 2008, Eliwa et al., 2021, Hagihara et al., 2013, Tunc-Ozcan et al., 2019). In support of this view, complementary evidence exists to suggest that AHN plays a key role in buffering against stress synthesising data from both animal models (Anacker et al., 2018, Yam et al., 2019) and human *post-mortem* studies (Boldrini et al., 2019). For example, using *in vivo* calcium imaging to record neuronal activity from large cell populations in the ventral DG (vDG), it was demonstrated that newborn neurons in this region act to decrease in the activity of other stress-responsive neurons that are preferentially active during social defeat attacks by aggressor mice or while mice explore anxiogenic environments (Anacker et al., 2018). These effects on AHN and thus DG activity are necessary and sufficient for stress resilience, as silencing of the vDG confers resilience whereas excitation promotes susceptibility (Anacker et al., 2018). These data also resonate with the substantial body of evidence linking exposure to stressful life events and increased risk for psychiatric illness (Syed and Nemeroff, 2017, Costello et al., 2003, Sarason et al., 1978, Heim, 2000, Andrews et al., 1978). Collectively, these data suggest that the degree of AHN may be one critical factor in determining an individual’s level of either risk or resilience to developing psychophatology, particularly in the context of stress exposure.

It is also clear that AHN is remarkably sensitive to environmental influences, consistent with the evidence that 30–40% of neurodevelopmental disorder risk may be attributed to environmental factors (Cattane et al., 2020). Besides the aforementioned and well-documented impact of stress on AHN, human epidemiological studies have consistently reported associations between maternal immune activation (MIA, by either infectious or non-infectious stimuli) and increased risk for a range of psychiatric and potentially neurological disorders in the affected offsrping (Kępińska et al., 2020, Knuesel et al., 2014, Estes and McAllister, 2016). This has led to development of a whole field of research into prenatal programming, for example following MIA, which may “pre-set” an individual’s risk for any subsequent differences in cognition, emotion or even psychiatric illness later in life (Bilbo and Schwarz, 2012, Kempermann, 2019). It is clear however that the impact of MIA is variable and a significant proportion of offspring exposed to MIA do not develop any overt behavioiural or psychological illness, That said, it is plausible that the absence of any such psychopathology could simply reflect a latent insult that may be unmasked by exposure to other environmental risk factors, including stress exposure during vulnerable periods of brain maturation such as adolescence. Regardless, of either possibility, the mechanisms underling either risk or resilience following MIA exposure remain unclear. Synthesising the aforementioned evidence that links reductions in AHN with increased risk, whislt boosting AHN may drive resilience for affective and non-affective psychiatric disorders, we hypothesised that a reduction in AHN following MIA during pregnancy might be one important driver of risk and resilience in the affected offspring. To address this, we first summarise the evidence linking AHN and MIA independently to increased risk for psychiatric disorders. We then consider the evidence supporting MIA-dependent disruption of AHN and how this relates to behaviour, including priming the response to stress exposure. Although we find evidence to support a link between MIA and disrupted AHN, we highlight the need for studies to directly test our hypothesis using validated animal models exposed to MIA in order to answer two key questions; First, does AHN differ between susceptible and resilient offspring? Second, does reduced AHN following MIA mediate susceptibility to a second risk exposure, such as stress (Mueller et al., 2021), even in apparently MIA-resilient offspring (Mueller et al., 2021, Ranaei et al., 2020, Giovanoli et al., 2013)? Finally, we highlight that to test the relevance of such data for human pathology, there is a need for complementary studies using human induced pluripotent stem cells (hiPSC) to understand how immune stimulation that results in MIA may impact upon AHN using a human cellular model system, which would also offers the potential to test gene * environment interactions using patient-derived material.

### 1.1 Adult hippocampal neurogenesis and behavioural disorder

Current evidence suggests there are only two brain regions in which adult neurogenesis may be detected in the human brain: the subgranular zone (SGZ) of the DG in the hippocampus, and the subventricular zone (SVZ) adjacent to the lateral ventricles (Spalding et al., 2013, Boldrini et al., 2018, Kempermann and Gage, 1999) (Fig. 1b). The proliferation of neural stem/progenitor cells (NSC/NPCs), as well as their differentiation into granule neurons and integration into existing circuits requires complex processes (Toda et al., 2019). Regulatory signals from the neurogenic niche direct neural stem cells to proliferate, migrate, extend neurites, form synapses and integrate into existing circuits (Fig. 1b).

Dysregulation of any these processes, particularly at the NSC/NPC stage, has the potential to permanently alter neuronal circuits within the hippocampus (Toda et al., 2019). The capability of NPCs to proliferate and differentiate is integral to the downstream quality of AHN. Conditional ablation of the miR-17–92 cluster in nestin + NPCs (stages 1–3 of Fig. 1b), known to regulate their function, significantly reduced proliferation and differentiation of NPCs in the DG which had a corresponding detrimental effect on hippocampus-dependent behaviour (Pan et al., 2019). On the other hand, the direct genetic-driven expansion of NSCs in the DG of mice by overexpressing Cdk4/cyclinD1 counteracts the age-related decline in AHN (Berdugo-Vega et al., 2020). Therefore, exposure to any entities, including MIA, capable of disturbing the essential function of the NSC/NPC pool has the potential to alter an individual’s capacity for AHN (Fig. 1b). Hence, one potential mechanism contributing to the emergence of psychiatric disorders could be the disruption of AHN by MIA, which may by itself lead to increased risk for psychopathology later life, or create a “latent insult” that may be subsequently unmasked by additional second risk exposure, such as stress (Fig. 1) (Toda et al., 2019, Kang et al., 2016, Anacker, 2014, Gergues et al., 2018).

### 1.2 Maternal immune activation

Maternal immune activation (MIA) from both infectious and non-infectious sources contributes to a significant environmental risk to neurodevelopmental sequelae (Meyer, 2019). Epidemiological studies have consistently linked MIA and higher levels of inflammatory markers to increased risk for psychiatric disorders with a putative neurodevelopmental origin in the affected offspring, including schizophrenia (SZ) and autism spectrum disorder (ASD) (Kępińska et al., 2020, Knuesel et al., 2014, Estes and McAllister, 2016). Causality for these associations is provided by “one-hit” animal models for MIA in which a single immune challenge is administered to the pregnant dam at key stages of gestation. Data from such models confirms that such exposure to immune activation during critical periods of brain development in gestation leads to abnormal trajectories of brain development (Piontkewitz et al., 2011, Crum, 2017) and the emergence of behavioural disruption in adulthood, including deficits in social interaction, cognition and so on (Meyer, 2019, Meyer, 2014). As such, the biochemical prenatal maternal response to such environmental factors, both infectious and non-infectious, represents a risk factor capable of disrupting foetal neurodevelopment. Several birth cohort studies implicate increased psychiatric disorder risk in offspring with prenatal stress (Malaspina et al., 2008, Brown and Conway, 2019), such as bereavement (Huttunen, 1978) and socio-economic disadvantage (Gilman et al.,). Except for specific viral infections with maternal-foetus vertical transmission such as Zika, Rubella and Human Cytomegalovirus (HCMV) (Claus et al., 2020), it is now generally accepted that most infectious MIA cases do not confer psychiatric risk by direct infection of the foetus via the placenta, but rather a systemic maternal immune response influencing neurodevelopment (Meyer, 2014).

In order to identify which components of the maternal immune response are central to the effect of MIA, birth cohort studies have examined links between fluctuations in specific cytokines in maternal serum and the degree to which offspring experience any psychopathology. For example, Allswede and colleagues reported increased concentrations of the classical pro-inflammatory cytokines TNFα, IL-1β, and IL-6 in the maternal serum of offspring who developed psychosis, suggesting this effect is greatest when the elevations in these cytokines are present during the weeks 7–20 of pregnancy (Allswede et al., 2020). Hence, proxy markers of early prenatal infection may be associated with a greater risk for psychosis in the offspring, demonstrating the developing brain’s increased sensitivity to environmental factors that can alter neurodevelopmental processes (Knuesel et al., 2014, Allswede et al., 2020, Adam, 2012). Further supporting this view, a recent human study by Goldstein and colleagues indicated that a higher ratio of TNFα:IL-10 in maternal serum correlated with sex-depedent increases in hippocampal activity and decreases in hypothalamus-hippocampus connectivity, as measured by functional magnetic resonance imaging (MRI) during response to stressful stimuli in the offspring even to the age of 45 years (Goldstein et al., 2021). Additionally, increased maternal IL-6 serum concentration correlated with decreased hippocampal acitivty in females only (Goldstein et al., 2021). These remarkable findings highlight that MIA can have long-lasting effects on stress circuitry in the brain, consistent with the idea of a “latent insult” such that MIA exposure may pre-set an increased sensitivity to negative stressful events in later life by disrupting the development of neural circuits linked to stress responses (Goldstein et al., 2021). In further support of this view, several recent studies have found a clear link between the concentration of circulating IL-6 in maternal serum with abnormal brain structural and functional connectivity, as measured by MRI and cognitive development and externalising behaviours in the offspring (Rasmussen et al., 2019, Rudolph et al., 2018, Graham et al., 2018). Furthermore, the mean maternal concentration of IL-6 can be inferred from offspring functional connectivity patterns, derived from MRI data in the newborn brain (Rudolph et al., 2018).

In further support of maternal IL-6′s crucial role in foetal development, an *ex vivo* isolated cotyledon human placental perfusion model indicated IL-6 to transfer bidirectionally between mother and foetus, but not IL-1α or TNFα (Zaretsky et al., 2004). Furthermore, a single prenatal injection of IL-6 to pregnant rat dams on gestational day (GD) 12.5 leads to similar behavioural dysfunctions in the offspring as induced following exposure of pregnant dams to either influenza virus or the toll-like receptor 3 (TLR3) agonist Poly I:C, suggesting a key role for this cytokine (Smith et al., 2007). Behavioural and molecular phenotypes in this mouse model are also ameliorated by administration of IL-6 neutralising antibodies, or if Poly (I:C) is administered to dams lacking the IL-6 receptor, confirming the importance of this cytokine (Smith et al., 2007). One important pro-inflammatory function of IL-6 is the promotion of T_H_17 cell differentiation, from which cells IL-17A is released (Lee et al., 2012). Notably, administration of IL-17 alone to pregnant mice also leads to behavioural deficits in the offspring which have relevance for both ASD and SZ (Choi et al., 2016). Finally, the response to MIA by maternal and neonatal cytokine serum concentrations is proposed to be genetically predetermined, after foetal loci for immune function were found to independently influence maternal immune mediators in a genome-wide association study (Traglia et al., 2018). Collectively, these data support the view that specific cytokines play a role in increasing risk for neurodevelopmental disorders by influencing neurodevelopment, as predetermined by genetic factors.

Nevertheless, epidemiology also provides evidence that prenatally exposed children can show resilience to neurodevelopmental deficits and remain healthy through adulthood (Meyer, 2019). Alternatively, consistent with the idea that exposure to MIA may predispose children to develop psychopathology in adulthood, that only emerges after additional environmental “hits” such as exposure to stress or drug abuse (Meyer, 2019, Bayer et al., 1999, Giovanoli et al., 2015). The interplay between offspring susceptibility and resilience to MIA is now being considered; under the notion that risk factors reach a threshold level within a vulnerable period of development, prior to precipitation of neurodevelopmental phenotypes (Meyer, 2019, Mueller et al., 2021).

These data raise important questions as to the mechanisms that define either susceptibility or resilience following exposure to pre- and post-natal environmental risk factors such as MIA and how this may prime suseceptibility to additional second hits such as stress. The immune system is known to play an essential role in the communication between the external environment and internal physiological and biochemical states (Rook, 2013). Hence, alterations to such physiological and biochemical states could subsequently mediate behavioural states via changes in the hippocampal neurogenic environmental niche. Given the evidence that AHN promotes resilience to stress (Anacker et al., 2018, Boldrini et al., 2019), it is reasonable to hypothesise that one mechanism by which MIA could prime offspring to be more sensitive to a post-natal stress exposure is through disruption of AHN, by creation of a “latent” insult. Such prenatal programming may therefore “pre-set” an individual’s risk for any subsequent behavioural disorder or psychiatric illness (Fig. 1a). But, what evidence is there that MIA disrupts AHN? In the following section, we will therefore consider the mechanisms by which MIA prenatally primes suppression of adult hippocampal neurogenesis, increasing susceptibility for psychopathology in adulthood.

## 2 Prenatal immune challenge alters hippocampal neurogenesis

Studies on adult hippocampal neurogenesis in the context of MIA have to date, largely been confined to “one-hit” animal models or *in vitro* cellular systems. Here we define a “one-hit” model as a single exposure to a pathogen, immunostimulant or specific cytokine. As previously reviewed (Brown and Meyer, 2018, Musaelyan et al., 2014), hippocampal neurogenesis in MIA models is usually investigated by exposing a pregnant animal or *in vitro* cultures to an immunostimulant, followed by assessment of behavioural, neurochemical, neuroanatomic, and neurophysiologic endophenotypes of relevance to brain disorders associated with impaired hippocampal neurogenesis in the offspring or using *in vitro* cell cultures (Musaelyan et al., 2014). Various immunostimulants have been used and include viral infection (e.g. Zika, human cytomegalovirus and porcine reproductive and respiratory syndrome virus (PRRSV) (Antonson et al., 2018, Dang et al., 2016, Meyer, 2006, Sun et al., 2020), viral or bacterial infection mimetics such as the toll-like receptor (TLR) 3 and 4 agonists Poly I:C and LPS respectively (Kępińska et al., 2020, Meyer, 2006, Musaelyan et al., 2014), or exposure to specific pro-inflammatory cytokines, such as IL-6, TNFα or IL-1β (Seguin et al., 2009). The following section will review recent works that incorporate such methods to investigate how MIA may supresses AHN either following the initial immue exposure, or as a consequence a “primed” response to subsequent immune activation following, for example, stress exposure.

### 2.1 The influence of MIA on AHN

There is evidence to suggest that a single, prenatal exposure to a “one-hit” single dose (5–20 mg/kg) Poly I:C, LPS or PRRSV exposure is sufficient to impair hippocampal neurogenesis in adulthood (Musaelyan et al., 2014). Test metrics used to quantify behaviour in animal MIA model offspring have been reported to correlate linearly with deficits in hippocampal neurogenesis in both adolescence and adulthood (Table 1). Both anxiety- and depression-related behaviours are commonly identified using Y-maze, prepulse inhibition (PPI), Morris water maze, open field, sensorimotor gating and motor coordination tests, in which MIA offspring perform significantly worse compared to control offspring (Depino, 2015, Li et al., 2020, Mattei et al., 2014, Sheu et al., 2019, Wolf et al., 2011, Zhang and van Praag, 2015, Zhao et al., 2019). Such deficits in fear-, anxiety-, and stress-related behaviour would be consistent with hippocampal dysfunction, including deficits in AHN, since the hippocampus is considered to be responsible, at least in part, for these behaviours (Bartsch and Wulff, 2015).

**Table 1:** MIA reduces hippocampal neurogenesis correlating with behavioural deficits. Evidenced by animal models from the last decade updated from (Musaelyan et al., 2014).

Reference	Animal	Stimulation	Timing	Age of offspring	Behavioural Effect	Effect on Neurogenesis	Phenotype rescued by
(Li et al., 2020)	Rats	Poly I:C 10 mg/kg	GD9.5	PND40	No effect was seen.	No effect on ERK1/2 phosphorylation. Increased total NFM immunoreactivity.	N/A
				PND60	Deficiencies in Y-maze entries, time spent in novel arm and PPI performance.	Reduced ERK1/2 phosphorylation. No change in total NFM immunoreactivity, but distribution decreased in the CA3 region and increased in DG.	
(Sheu et al., 2019)	Mice	Poly I:C 5 mg/kg	GD17	PND42	No effect was seen.	No effect seen.	N/A
				PND63	Decreased time in central zone, sucrose preference, increased immobility and latency to feed.	Reduced proliferation and aberrant dendritic development of newly generated neurons.	
				PND84			
(Zhao et al., 2019)	Rats	Poly I:C 10 mg/kg	GD18	PND27-28	Abnormal behaviours in Morris water maze and elevated plus maze	Reduced proliferation and differentiation of SGZ cells.	PPARγ agonist Pioglitazone improved hippocampal-dependent spatial learning, memory and neurogenesis.
(Antonson et al., 2018)	Pigs	PRRSV	GD76	GD111 (3 days before suspected birth)	N/A	No change in the total number of hippocampal proliferative cells. Decreased brain weight and reduced neuronal number in DG. GFAP + density and expression increased.	N/A
(Mouihate, 2016)	Rats	LPS 100 µg/kg	GD15	PND45	N/A	Decreased newly born neurons in DG but not SVZ.	IL-6 blocking antibody and cortisone receptor blocker RU-486.
(Depino, 2015)	Mice	LPS 25 µg/kg	GD9	PND56-70	Increased anxiety- and depression-related behaviours	Decreased NA and 5-HT in the hippocampus. No difference in proliferation and differentiation in DG but decreased Reelin in the hippocampus.	N/A
(Zhang and van Praag, 2015)	Mice	Poly I:C 5 mg/kg	GD15	PND90	Deficiencies in PPI, spatial maze and motor coordination test plus increased depression-related behaviours	Reduced DG volume and PV + interneurons. Hippocampal neurons had increased input resistance, lower current threshold and decreased action potential number.	N/A
(Mattei et al., 2014)	Rats	Poly I:C 4 mg/kg	GD15	PND90-128	Deficient in PPI test	Decreased proliferation and net-neurogenesis in the DG. Decrease in microglia Iba1 reactivity.	Minocycline rescued both neurogenic and behavioural deficits, but not microglial.
(Melnik et al., 2012)	Mice	Poly I:C 16 kg/ml	GD15	PND60	N/A	Reduced proliferation of NPCs in DG.	N/A
(Piontkewitz et al., 2012)	Rats	Poly I:C 4 mg/kg	GD15	PND100	N/A	Decreased early (PND14-36) proliferation in DG but did not affect later (PND 77–79) differentiation of mature neurons in the DG.	Risperidone increased number of proliferating cells, percentage of differentiating cells and prevented a decrease in PV + HP interneurons.
(Wolf et al., 2011)	Mice	Poly I:C 5 mg/kg	GD15	PND60	Deficits in open field test and sensorimotor gating	Premature senescence, reduced telomere length and telomerase activity of NPCs in the DG.	Voluntary exercise increased telomerase activity but not telomere length, also rescuing behavioural and neurogenic phenotype.

In this context, *post-mortem* studies in “one-hit” animal models of MIA provide evidence for reduced proliferation of hippocampal NSC/NPCs and impaired differentiation as indicated by reduction of cells expressing the proliferation marker BrdU and the immature neuron marker DCX (Mattei et al., 2014, Melnik et al., 2012, Mouihate, 2016, Piontkewitz et al., 2012, Sheu et al., 2019, Wolf et al., 2011, Zhao et al., 2019). These data confirm that the hippocampal pool of NSC/NPCs in adulthood is vulnerable to prenatal immune challenge, resulting in impaired proliferation and differentiation (Fig. 1b). The use of *post-mortem* animal studies at adolescent and adult timepoints alone, however, does not aid an understanding of the molecular basis by which MIA influences hippocampal neurogenesis at the point of prenatal exposure. Additional evidence from early, prenatal time-points relevant to prenatal risk exposure is needed to examine the acute, proximal effect of MIA on hippocampal neurogenesis.

#### 2.1.1 Effects of MIA on AHN mediated by cytokines

The maternal cytokine-related immunological response to infection is one essential mechanism by which MIA confers increased psychiatric disorder risk in offspring (Allswede et al., 2020, GILMORE and FREDRIKJARSKOG, 1997). Various cytokines have been determined as essential in conferring the long-lasting negative impact of TLR4 and TLR3 prenatal activation in animal models (Mouihate, 2016, Smith et al., 2007). As already stated, LPS and Poly I:C do not cross the foetal-placental barrier, but pro-inflammatory cytokines transduce their effects in animal MIA models (Ashdown et al., 2006, Zaretsky et al., 2004). The systemic maternal cytokine response to MIA risk factors therefore has the potential to influence embryonic hippocampal neurogenesis.

Interferons (IFN) are produced by the innate immune system in response to environmental insults such as viral infection and are known to induce altered microglial phenotype and behavioural abnormalities Poly I:C induced MIA mice offspring (Ben-Yehuda et al., 2020). Using an immortalised human hippocampal progenitor cell line Borsini and colleagues demonstrated physiologically relevant concentrations of IFNα decreased neurogenesis and increased apoptosis at high concentrations (Borsini et al., 2018). Oxidative stress, immune response, neuronal formation and cell death regulation pathways were implicated in the action of IFNα on the human hippocampal cells (Borsini et al., 2018). Furthermore, chronic human IFNα treatment has been shown to reduce AHN and impair adaptive behaviour in adult common marmosets (Kaneko et al., 2020). MIA-dependent release of interferons can therefore acutely dysregulate neuronal maturation of NSC/NPCs in hippocampal regions.

Aside from interferons, maternal IL-6 is considered a sensor, effector and transducer of MIA (Seguin et al., 2009, Entringer et al., 2015) and also plays a key role in the regulation of neurogenesis (Erta et al., 2012). In the aforementioned study by Borsini and colleagues, dual administration of IFNα with IL-6 produced a synergistic effect on hippocampal progenitor cell death. Aquaporin 4 was downregulated, suggesting an imbalance in the cell’s homeostatic capacity (Borsini et al., 2018). Offspring from LPS-challenged pregnant rat dams had a reduced number of newly born neurons in the DG in adulthood, as monitored by a decrease in the number of DCX and T-box brain protein-2 (TBR2) expressing NPCs (stages 2–4 of Fig. 1b) (Mouihate, 2016). Nevertheless, when LPS was co-administered with an IL-6 blocking antibody, the observed reduction of neurogenesis was salvaged suggesting an essential role for IL-6 in mediating changes in NSC/NPC differentiation in the DG (Mouihate, 2016). In a 3D neural differentiation model, referred to by the authors as “neural aggregates” that resembled neurospheres, IL-6 exposure did not affect neural aggregate size (Zuiki et al., 2017). Instead, it decreased the more mature TBR1^+^/CTIP2^+^ cell population’s area ratio (stage 5 of Fig. 1b) without changing the proportion of latter born SATB2^+^ neurons (stage 4 of Fig. 1b), suggesting a distorted cell fate differentiation or increased NPC-specific apoptosis (Zuiki et al., 2017). Besides IFN, IL-6 can therefore additionally acutely interfere with hippocampal neuron maturation.

Certainly, the influence of MIA on AHN is not as a result of the acute release of IFN and IL-6 alone. For example, IL-6 has the additional potential to regulate DNA methylation in human-derived cells (Hodge et al., 2001), which itself has been shown to play a role in AHN (Jessop and Toledo-Rodriguez, 2018, Richetto et al., 2017, Zhang et al., 2013). Consequently, systemic cytokine signalling at an early vulnerable age of neurodevelopment provides MIA with a possibility to leave a dormant mark on neurogenic and cell survival programs, which in turn could alter neurodevelopmental and neurogenic trajectories to make an individual more or less susceptible to stressors in later life.

#### 2.1.2 Effects of MIA on AHN mediated by changes in hippocampal neuron activity

Given that AHN is essential to the on-going formation and refinement of hippocampal neurocircuitry throughout adulthood, it is not surprising that the disruption of this process may result in the onset of atypical behaviours (Anacker and Hen, 2017). As reviewed elsewhere, new-born adult neurons generated by AHN inhibit the activity of mature granule cells in the DG, which contributes to information encoding (Anacker and Hen, 2017). Thus, AHN affects reversal learning and cognitive flexibility via the regulation of DG neuron activity (Anacker and Hen, 2017). As previously mentioned, inhibition of these new-born adult neurons in the vDG of rodent models increases the susceptibility to stressors in later life and vice versa to confer resilience to chronic stress (Tunc-Ozcan et al., 2019, Anacker et al., 2018). Therefore, the disruption of hippocampal neuron functioning by MIA could render an individual more susceptible to stress exposure as their adult born neurons are less capable of regulating a hippocampal stress circuit, which AHN directly regulates (Anacker et al., 2018, Patrich et al., 2016). Patrich and colleagues demonstrated in primary hippocampal culture from postnatal day (PND) 0–2 rodents that MIA exposed offspring had a considerably lower intrinsic excitability and stronger spike frequency adaptation (Patrich et al., 2016). In *post-mortem* brain tissue from the same study, two-weeks old MIA exposed rodent offspring had a lower intrinsic excitability in CA1 pyramidal neurons (Patrich et al., 2016). Hence, MIA has the potential to dysregulate hippocampal neuron function in early life (Patrich et al., 2016), potentially leaving an individual more susceptible to stress, given their reduced capacity to regulate a hippocampal stress circuit (Anacker and Hen, 2017, Patrich et al., 2016). The combination of reduced AHN plus the potential for MIA-dependent dysregulated hippocampal neuronal function may therefore leave an individual predisposed to behavioural disorder and/or psychiatric illness after stress exposure.

### 2.2 Effect of MIA on AHN via immune “priming”

Typically, the age of SZ onset is around late adolescence to early adulthood (Häfner et al., 1994). If such disorders were caused by MIA *per se*, the onset of psychiatric disorders in some cases is therefore latent rather than immediate. A handful of “one-hit” MIA models which investigated more than one postnatal timepoint reported that deficiencies in AHN post MIA occur in an age-dependent manner (Antonson et al., 2018, Depino, 2015, Li et al., 2020, Sheu et al., 2019). Sheu and colleagues determined gestation day (GD)17 Poly I:C exposed mice offspring at 6-weeks of age to have no behavioural phenotype or differences in AHN as compared to control offspring (Sheu et al., 2019). Nevertheless, after 9- and 12-weeks postpartum, the MIA offspring developed anxiety- and depressive-like behaviours that paralleled reduced proliferation and aberrant dendritic development of newly generated neurons in the DG, indicating a latent onset of these phenotypes in adulthood (Zhao et al., 2019). These data raise the question; by what means could the effects of MIA on AHN lie dormant until adolescence or adulthood? The latent onset of deficits in behaviour and AHN in adulthood suggests risk priming, whereby offspring may be more susceptible to future stress, which could unmask latent behavioural dysfunction and/or psychopathology. Conceivable mechanisms by which such immune priming following MIA exposure may affect AHN, and thus some behaviours, are presented as follows.

#### 2.2.1 Microglia

Multiple lines of evidence from human genetics, *post-mortem*, neuroimaging and peripheral biomarker studies implicate the innate immune system and particularly microglia in the pathophysiology of neuropsychiatric disorders (Mondelli et al., 2017). As the resident CNS myeloid cells, microglia play critical roles in shaping the central immune response to maintain homeostasis (Hanger et al., 2020). The disturbance of healthy microglial function by MIA during foetal brain development could, in turn, disrupt essential processes required for healthy foetal or adult neurogenesis and neural circuit formation (Knuesel et al., 2014). Considering that MIA impacts microglial neurodevelopmental function most potently in early life (Matcovitch-Natan et al., 2016), but also primes the immune system to respond more strongly to stress (Giovanoli et al., 2013), dysfunctional microglia could cause abnormal AHN. The influence of MIA activated microglia could perhaps sensitise the individual to greater immune responses following stress exposure, thus leading to a latent effect on AHN to trigger pathology in later life.

Microglia are highly plastic cells, which can assume a range of functional and morphological states, resulting in significant intra- and inter-regional heterogeneity in the brain (Hanger et al., 2020, Sharma and Tremblay, 2020). Microglia polarised to a pro-inflammatory state release reactive oxygen species, complement proteins, proteinases and pro-inflammatory cytokines such as IL-6, TNFα, IL-1β, which attract other immune cells, such as macrophages, dendritic cells and lymphocytic T-cells, facilitating their entry into the brain across the blood–brain barrier and promoting phagocytosis of foreign agents or neurotoxic cellular debris (Barres, 2008, Hanger et al., 2020). These actions can impede the neurogenic cascade during chronic stress (Sierra et al., 2014). In contrast to a pro-inflammatory phenotype, microglia can also present anti-inflammatory phenotypes, secreting cytokines, chemokines and trophic factors to promote repair and re-establish homeostasis, essential for encouraging neurogenesis. A prolonged pro-inflammatory response can be detrimental to nearby neurons, given the molecules’ potential cytotoxic nature. As a result, the damaging impact of pro-inflammatory microglia on neurogenesis is well supported (Ekdahl et al., 2009, Knuesel et al., 2014, Sierra et al., 2014). Importantly, microglia are strongly implicated in influencing AHN by regulating neuronal proliferation and differentiation, as well as synaptic connections, via phagocytosis of apoptotic new-born cells, secretion of neurotrophic factors and surveillance communication to nearby neurons by CX_3_CR1/CX_3_CL1 signalling (Gemma and Bachstetter, 2013). Therefore, microglia are important mediators in the modulation of cognitive and behavioural functions downstream of AHN.

Encouraging microglia towards an anti-inflammatory state benefits AHN (Han et al., 2019, Monje et al., 2003, Zhang et al., 2021). In the context of an immune response, Monje and colleagues revealed adult rodents exposed to LPS had an increased number of activated microglia in the DG and impaired AHN (Monje et al., 2003). The increase in activated microglia corresponded with increased cytokine levels, including IL-6. This immune response mediated AHN impairment as demonstrated by the fact that Indomethacin, a nonsteroidal anti-inflammatory, rescued the AHN deficit (Monje et al., 2003). Although the LPS stimulation during adulthood is not physiologically relevant to MIA, these data are consistent with a view that microglial function supports typical AHN. Furthermore, in a “two-hit” early adversity mouse model by Han and colleagues whereby male offspring were separated from their mothers for 3 h a day through PND1-14 and then a second stress 3 weeks after weaning, administration of the anti-inflammatory drug minocycline between stresses reversed the pro-inflammatory markers and microglia transition states that correlated with altered hippocampal neurogenesis (Han et al., 2019). Finally, Zhang and colleages identified the importance of anti-inflammatory microglia after knocking down the expression of the anti-inflammatory marker IL-4 receptor (IL4R) caused a reduction of hippocampal neurogenesis and increases susceptibility to chronic mild stress in mice, and vice versa when overexpressing IL-4 (Zhang et al., 2021). Interestingly, IL-4 expression in MIA-exposed offspring is reduced in the hippocampus up to PND7 compared to control offspring, meaning a potential for unchecked pro-inflammatory microglia to excert their effects on hippocampal neurogenesis during this time (Garay et al., 2013).

In recent work, Zhao and colleagues examined the potential beneficial effects of pioglitazone given on days PND21-27 to the offspring of pregnant rats exposed to Poly I:C or saline GD18 (Zhao et al., 2019). Pioglitazone activates the peroxisome proliferator-activated receptor gamma (PPARγ), which is expressed in human neurons, microglia, oligodendrocytes, endothelial cells and astrocytes (Zhang et al., 2016). Prepubescent MIA-offspring (PND28) subsequently presented abnormal learning and memory behaviours, the severity of which were positively associated with impairment of hippocampal neurogenesis and increased microglial, but not astrocytic density, in the DG (Zhao et al., 2019). Pioglitazone treatment improved both hippocampal neurogenesis and hippocampal-dependent spatial learning in the exposed offspring. Implicating microglial involvement in the DG, pioglitazone treatment also reduced total microglial population and the number of microglial processes (Zhao et al., 2019). Transcript levels of pro-inflammatory cytokines TNFα, IL-1β, IL-6, CD68, iNOS and IFNγ were increased in the hippocampus of MIA offspring, but returned to control levels upon pioglitazone administration (Zhao et al., 2019). Using immunofluorescence co-localisation, the source of IL-6 was determined by Zhao and colleagues to be microglial cells, indicating the involvement of a microglial phenotype in reduced neurogenesis in the hippocampus (Zhao et al., 2019). Hence, promoting microglia towards an anti-inflammatory phenotype by PPARγ, activation, IL-4 signalling, minocycline and Indomethacin demonstrates pro-neurogenic effects, ameliorating cognitive impairments, anxiety-related behaviours and reduced AHN caused by a prenatal immune challenge (Han et al., 2019, Monje et al., 2003, Zhang et al., 2021, Zhao et al., 2019).

Nevertheless, many studies suggest that at the point that neonatal and postnatal periods are reached, MIA-induced microglial activation is resolved (Antonson et al., 2018, Giovanoli et al., 2015, Missault et al., 2014). Therefore, an acute pro-inflammatory microglia phenotype is unlikely to be the main mechanism by which MIA results in neurogenic deficiencies that are observed in neuropsychiatric disorders. Instead, MIA might induce immune memory in microglia without the overt longstanding phenotypic expression. For example, neonatal infection altered microglia response to immune challenge after the reexposure to LPS in adulthood (Bilbo, 2005). The reexposed animals showed greater and more prolonged increase in microglial marker mRNA expression and faster IL-1β upregulation in the hippocampus and cortex (Bilbo, 2005). This suggests that adverse prenatal or early life experiences inform microglia response to such challenges in adulthood. Such priming effect could in part be mediated through epigenetic modifications. Although insights on the epigenetic mechanisms of immune memory formation in microglia remain limited, changes in histone deacetylase (HDAC) expression and activity as well as repressive (H3K27me3) and transcriptionally active (H3K4me3) histone marks have been implicated in studies, exploring microglia priming to such stimuli as LPS or glioma-conditioned medium (Cao et al., 2015, Maleszewska et al., 2021, Martins-Ferreira et al., 2021, Schaafsma et al., 2015). This way altered microglial state by prenatal MIA challenge might make an offspring more susceptible to future stress, which, unbalanced by AHN dependent mechanisms of stress resilience, could thus increase their risk for psychiatric illness (Giovanoli et al., 2013).

#### 2.2.2 Telomeres

Telomere length has also long been associated with neurogenic disruption and SZ (Kempermann et al., 2008). Telomere length maintenance during mitosis is essential in cells with a high turnover rate, which NSCs and particularly NPCs have (Klapper et al., 2001). Mice deficient in telomerase (TERT), the enzyme that maintains telomere length, have shorter telomeres and disrupted SVZ neurogenesis with additional deficits in behaviour dependent on this part of the brain (Ferrón et al., 2004). Schizophrenic patients also have reduced TERT activity (Kempermann et al., 2008). Work by Wolf and colleagues identified premature NPC senescence in the DG correlated with reduced telomere length, telomerase activity and behavioural deficits in open field test and sensorimotor gating in offspring of a “one-hit” animal MIA model exposed to a single prenatal Poly I:C challenge on GD15 (Wolf et al., 2011). Except for telomerase activity, these phenotypes were rescued by voluntary exercise, a known hippocampal neurogenesis stimulant (Fabel and Kempermann, 2008, Wolf et al., 2011, Zang et al., 2017). This highlights the possibility that telomerase activity could be responsible for the disease-related decline of hippocampal neurogenesis. Telomere length is already reduced with age, irrespective of any atypical behaviour diagnosis or genetic risk for psychiatric disorders (Palmos et al., 2018, Palmos et al., 2020). MIA’s acceleration of this phenomenon only exacerbates the reduction of hippocampal neurogenesis with age. Nevertheless, the question remains; how does MIA exacerbate telomerase shortening to indirectly pre-set atypical AHN?

**Fig. 2:**
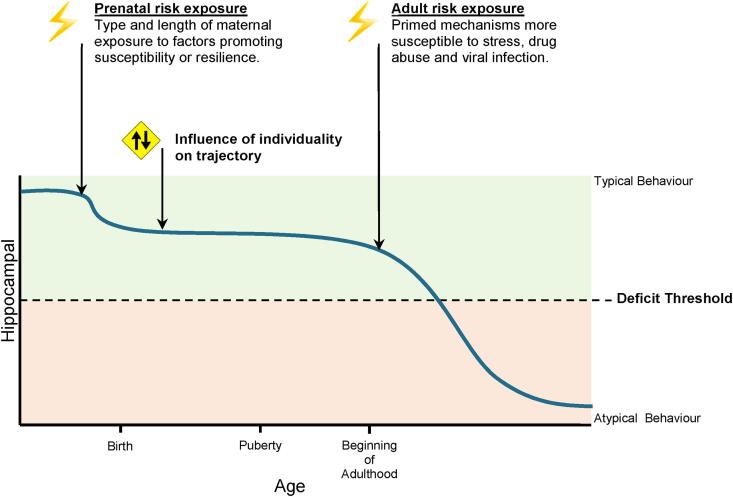
The trajectory to atypical hippocampal neurogenesis. Once hippocampal neurogenesis surpasses a threshold, indicated by the dotted line, behavioural deficits are precipitated. Prenatal priming and individuality can influence the susceptibility to factors that cause neurogenesis trajectory to pass this threshold. Axes are not to scale. Graphics created with BioRender.com.

In summary, AHN is significantly reduced in MIA-exposed adolescent and adult offspring. MIA has the potential to pre-set an alternative trajectory of an individual’s capacity for AHN, through numerous mechanisms, summarised in Fig. 2. The resulting AHN capacity of an individual may then mediate their resilience or susceptibility to a secondary stressor exposure, which in turn has the potential to precipitate behavioural disorder and psychiatric illness. It may be hypothesised that if this trajectory dips below a certain critical threshold, psychiatric illness becomes increasingly likely.

## 3 The knowledge gap, and how to bridge it

Integrating the aforementioned evidence, we can see MIA causes reduced AHN at adolescent and adult timepoints by numerous mechanisms. Evidence for whether MIA-disrupted AHN mediates either resilience or susceptibility in either the “one-hit” MIA model or, more likely, the “two-hit” MIA plus stress model is now needed. Very few, if any, studies have addressed AHN capacity after both initial prenatal MIA and secondary peripubertal stressor exposure. A complex interplay of multiple factors contributes to the susceptibility or resilience to behavioural disorder in MIA-exposed offspring (Meyer, 2019, Mueller et al., 2021). Mueller and colleagues recently demonstrated the metrics measuring behaviour, transcriptomics, brain networks and cytokine profiles in adult isogenic mice offspring exposed to Poly I:C prenatally on GD12 clustered into two groups of offspring that were either susceptible or resilient to behavioural disorder (Mueller et al., 2021). The fact that within-litter offspring were not equally affected by MIA holds particular relevance when concluding outcomes of “one-hit” MIA models.

**Fig. 3:**
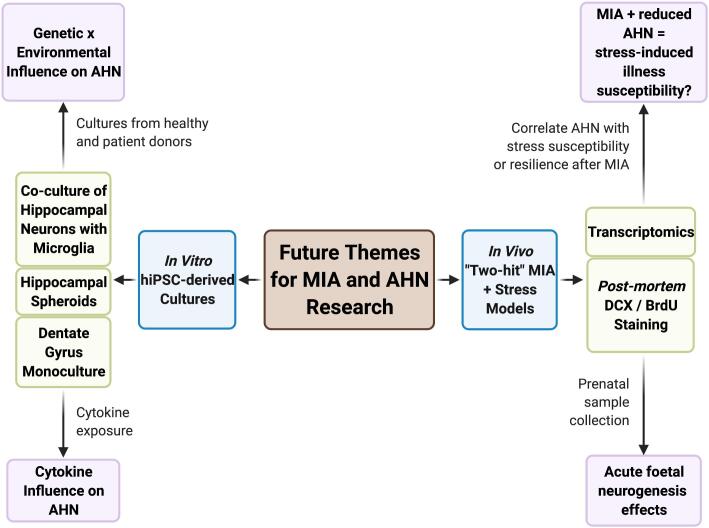
Future themes for MIA and AHN research. To fill the knowledge gap in how AHN actually mediates susceptibility to stressors after prenatal MIA exposure, we suggest following two themes of modelling: *in vivo* “two-hit” animal models incorporating both MIA and stress exposure; and *in vitro* hiPSC-derived patient material to physiologically represent human hippocampal neurogenesis under the direct influence of cytokines or indirect microglia-released cytokines.

Adult offspring defined as susceptible to MIA could also be characterised by increased circulating levels of pro-inflammatory cytokines, including TNFα, IL-1β, and IL-6 (Mueller et al., 2021). Whether these peripheral changes occur in the brain and if they are present from early development, however, remains to be determined. The impact, therefore, on an individual’s capacity for AHN could be affected by cytokine response and additional mechanisms to leave an offspring more or less vulnerable to stress exposure. In order to confirm the hypothesis that resilience and susceptibility to psychiatric illness is mediated by AHN following MIA, “two-hit” MIA plus stress animal models are essential to fill the knowledge gap (Fig. 3). Furthermore, our understanding of the extent to which atypical neurogenesis is provoked by MIA at the point of prenatal exposure in the developing embryonic hippocampus is incomplete. The use of human induced pluripotent stem cells could be used to bridge this knowledge gap, as well as translating the pre-collected animal data to a human context (Fig. 3).

### 3.1 Upgrading from “one-hit” to “two-hit” MIA models

The age at which neurodevelopment is vulnerable to risk is not exclusive to a single prenatal period, but also adolescence and adulthood (Meyer, 2019). Postnatal exposure to secondary risk factors, such as drug abuse, systemic infection and stress, can result in the onset of psychiatric disorders (Kępińska et al., 2020, Giovanoli et al., 2013, Meyer, 2019, Bayer et al., 1999). This phenomenon, known as the “two-hit” hypothesis, may explain why some psychiatric illness precipitate in late adolescence or adulthood (Bayer et al., 1999). Animal MIA models exposed to repeated or chronic low dose immune stimulants may be more physiologically relevant to encapsulate the intricate interplay of exposure to the multiple risk factors experienced by humans, as well as post-natal stress exposure. It is important to understand whether AHN plays a role in mediating the resilience or susceptibility to psychiatric illness onset after MIA exposure. Using “two-hit” animal models by incorporating both MIA prenatal insult with a secondary stressor, with subsequent investigation of potential differences in AHN, could aid in revealing the extent to which an individual’s capacity for AHN dictates their susceptibility to psychiatric illness.

Correspondingly, “two-hit” models could reveal crucial time periods in which boosting AHN in high-risk patients could act as a prophylactic against psychiatric disorder onset. In fact, returning to existing *post-mortem* hippocampal sections and transcriptomic datasets from previous datasets using either a “two-hit” MIA plus stress approach or identification of MIA-resilient and -susceptible groups (Mueller et al., 2021, Ranaei et al., 2020) could immediately answer some of the following questions. Does an individual’s capacity for AHN correlate with their susceptibility or resilience to behavioural disorder after prenatal MIA exposure? How would this kind of developmental design leave MIA models vulnerable to the risk of behavioural disorder in terms of risk priming? What are the implications for adult hippocampal neurogenic functionality after the combination of these types of stimuli?

### 3.2 hiPSC-Derived MIA models

Crucially, disparities in timing and dose of prenatal stimulation across comparative studies could be responsible for varying conclusions reached by animal MIA models. Variability in response to prenatal challenge originates from different rodent strains, gestational timing, dose, frequency, route of administration and batch of TLR3 or TLR4 agonists from different vendors (Mueller et al., 2019, Smolders et al., 2018). The molecular weight of Poly I:C can predict rat maternal IL-6 response and litter size, placental weight and male-specific reduction in foetal brain weights (Kowash et al., 2019). It is also vital to take into account both the timing of prenatal exposure and subsequent age of analysis, as variations in developmental timing can provide varying results since distinctive phases of pregnancy are susceptible to an assortment of environmental inputs at any one time (Allswede et al., 2020, Meyer, 2006). Consequently, questions surrounding the comparison of animal MIA models from multiple labs are raised, highlighting the importance of stimulant quality control for successful animal MIA models and accurate reporting of all model parameters, including housing and other variables (Kentner et al., 2019, Mueller et al., 2019).

Despite these caveats, it is striking to note the convergence of data across studies in MIA animal models providing converging evidence that AHN is reduced in adulthood following prenatal MIA exposure. Nevertheless, even though AHN is conserved across species, there are species-related differences that have to be taken into account (Kuhn et al., 2018). Animal brain development differs significantly from humans in terms of gene regulation networks and cellular proliferation pathways (Rockowitz and Zheng, 2015, Yokoyama et al., 2014). Hence, it cannot be assumed that the conclusions reached in animal MIA models will directly translate to human physiology. It is therefore important to complement this evidence base from animal models by using human based cellular models. Cell culture models are valuable tools to study biological mechanisms underlying health and disease in a controlled environment. It is important to keep in mind however, that cellular genotype plays a major role in the phenotypes observed *in vitro*, including, in the context of this review, shaping the cellular response to immune stimuluation *in vitro*. Of note then, the prevalence of subtle genetic variations in cell lines are not routinley characterised and taken into account when interpreting the data from *in vitro* studies of the effects of immune activation on neurogenesis. For example, the human hippocampal progenitor cell line (HPC0A07/03C) is frequently used as an *in vitro* model to study hippocampal neurogenesis (HN) (Borsini et al., 2018). A recent analysis of single nucleotide polymorphisms (SNPs) relevant to inflammation-related genes suggests that all stages of hippocampal neurogenesis may be negatively affected due to the genetic makeup of HPC0A07/03C cells following immune stimulation (Lee, 2021). Whilst these findings remain to be tested and validated experimentally, these data suggest a view that *in vitro* studies using such progenitor cell lines need to control for the genetic background of the cell line, since this could either mask or exacerbate findings (Lee, 2021). Taking this into account, we make the case for using human patient-derived material as a *complement* to animal and other cellular MIA modelling, specifically, hiPSCs. These cells offer many possibilities, as they can be differentiated into hippocampal granule neuron-like cells *in vitro* (Yu et al., 2014), as well as into different other neural or glial lineages and may be studied under either normal conditions or the influence of disease-causing immune exposure (Hoffman et al., 2019). Critically, the use of patient-derived material confers the important advantage of incorporating the various levels of polygenic risk in addition to environmental risk to further model the molecular mechanisms of MIA, particularly since many psychiatric illnesses share common genetic risk variants (O'Donovan and Owen, 2016). Importantly, in addition to using patient-derived cell lines, the use of genome editing may allow to investigate the role of specific genes in the effect of MIA on AHN on an isogenic background. Elucidating the genetic background of hiPSC lines is however still an important and necessary control.

As discussed in previous sections, epigenetic modifications might mediate the long-term effects of MIA and likely shape the cellular responses following subsequent exposures later in life, in line with “two-hit” hypothesis. Importantly, reservations do exist as to whether hiPSCs are a suitable model to assess epigenetic effects due to major epigenetic remodelling involved in their generation (Perrera and Martello, 2019). During differentiation, hiPSC-derived cells do undergo epigenetic remodelling (Bachmann et al., 2021, Su et al., 2021), and genome-wide DNA methylation patterns and gene expression patterns are preserved between neurons, derived from hiPSCs and human embryonic stem cells (de Boni, 2018). This provides confidence that hiPSC-derived cells would undergo MIA-associated epigenetic changes in similar manner as seen in *in vivo* models, thus making them a potentially useful model to investigate this. However, care should be taken to establish the baseline epigenetic signatures of the derived cell types to ensure valid like-with-like comparisons upon exposure to environmental stimuli.

A key barrier to the success of hiPSC modelling however, is the need to be able differentiate thse cells successfully into the relevant cell types, but also have the ability to capture interactions between such immature neurons with non-neuronal cells, such as microglia and/or endothelial and ependymal cells which form brain barriers *in vivo*, in a dish. Recently, Pomeshchik and colleagues have published a novel method of deriving hippocampal spheroids (HS) from hiPSCs which allows investigation of not only hippocampal neurons but also multiple stages of AHN, as well as other cell types within the neurogenic niche at a cellular and molecular level (Pomeshchik et al., 2020). While these hiPSC-derived HS have the advantage of incorporating multiple cell types, including both glia and neurons, the subsequent advancement would be to develop a model that features regional organisation of the hippocampus which has been attempted in part by a 2D model culturing both DG neurons and CA3 neurons in microfluidic chambers (Sarkar, 2018). To uncover the molecular effect of immune challenge on developing foetal hippocampal neurons, hiPSC-derived HS or NPC cultures could be exposed to an immune challenge from infectious viral particles, non-infectious mimic or cytokines as mirrored by studies (Claus et al., 2020, Dang et al., 2016, Sun et al., 2020, Warre-Cornish et al., 2020). As a further proof of concept investigated in hiPSC-derived cortical cultures, treatment of developing cortical NPCs with IFNγ induced widespread transcriptional changes that overlap with gene expression signatures measured in human *post-mortem* brain tissue from ASD and SZ patients, as well as increased neurite outgrowth, a hallmark phenotype of hiPSC-neurons derived from donors with genetic risk factors for ASD such as Shank-3 deletion (Schafer et al., 2019, Warre-Cornish et al., 2020).

The detrimental direct and indirect effects of cytokines released from activated microglia on AHN are clearly important to the mechanism by which an individual becomes predisposed to psychiatric illness after MIA exposure. These mechanisms can be adequately modelled at a cellular and molecular level either using organoids or co-culture of hiPSC-derived hippocampal neurons or NPCs and microglia. The interaction of microglia with hippocampal neurogenesis *in vitro* could be modelled in a number of ways, including monoculture conditioned media experiments, co-culture by physical separation, 2D, 3D-scaffold or even 3D-organoid culture (Hedegaard et al., 2020, Pellegrini et al., 2020). Such organoid cultures are becoming so complex, that they are able to derive the correct morphology, maturation and function of the choroid plexus (Pellegrini et al., 2020). Similar to the aforementioned studies, cultures could then be exposed to a singular or mixtures of cytokines to reveal the molecular mechanisms by which microglia exert a direct or indirect effect on hippocampal neurogenesis. Yet, clearly, different hiPSC model systems will be required to address the complex interactions between MIA and AHN *in vitro*, depending on the experimental question.

While hiPSC-derived models are highly suitable to study developmental effects, the relative immaturity characteristics of hiPSC-derived cultures make it difficult to assess the molecular effect of MIA on hippocampal neurogenesis during adulthood. To overcome this technical issue, it might be worth artificially ageing the *in vitro* model (Mertens et al., 2018). This can be done using various strategies; by manipulation of age-related genes or telomerases (Cozzi et al., 2019, Liang, 2020, Miller et al., 2013, Vera et al., 2016), by exposure to x-ray irradiation (Carlessi et al., 2009, Schneider et al., 2013, Schneider and Zheng, 2014, Zhu et al., 2019, Limbad et al., 2020), hydroxyurea (Daniele et al., 2016, de Lucia et al., 2020, Dong et al., 2014), repeated passaging (de Lucia et al., 2020, Han et al., 2021, Liu et al., 2012, Zhu et al., 2019) or long term culture (Daniele et al., 2020, Bigagli et al., 2016) to induce senescence-like changes into the hiPSC-derived cultures for a more aged-like phenotype.

## 4 Conclusion

Reduced AHN is linked closely to risk and resilience for psychiatric illness. Animal and *in vitro* MIA models indicate prenatal immune exposure reduces AHN through multiple mechanisms. We advance the hypothesis that, mediated by AHN, MIA renders an individual susceptible to additional “hits” such as stress. Combined this may be one important mechanism by which prenatal programming predisposes an individual’s risk for psychiatric illnesses. Once the accumulation of risk factors promoting susceptibility to hippocampal neurogenic deficits reaches a threshold, atypical behaviour ensues. However, the exact molecular mechanism and timing by which MIA primes latent dysfunction in hippocampal neurogenesis, plus the role of genetic interaction, remains to be found. Complementary research lines exposing animals to a “two-hit” MIA plus stressor experience and complimentary studies using relevant hiPSC-derived cultures exposed to cytokines are required to confirm or refute this hypothesis. Such studies have the potential to also uncover the mechanisms by which MIA confers susceptibility or resilience to psychiatric disorders, and the extent to which AHN mediates this risk.

## Declaration of Competing Interest

The authors declare that they have no known competing financial interests or personal relationships that could have appeared to influence the work reported in this paper.
